# Antimicrobial, antioxidant and cytotoxic activities of the leaf and stem extracts of *Carissa bispinosa* used for dental health care

**DOI:** 10.1186/s12906-023-04308-x

**Published:** 2023-12-15

**Authors:** Wanda Shekwa, Tsolanku Sidney Maliehe, Peter Masoko

**Affiliations:** Department of Biochemistry, Microbiology and Biotechnology, University of Limpopo, Private bag X1106, Sovenga, 0727 South Africa

**Keywords:** *Carissa Bispinosa*, Phytochemicals, Antimicrobial activity, Antioxidant activity, Cytotoxicity

## Abstract

**Background:**

*Carissa bispinosa* (L.) Desf. ex Brenan is one of the plants used traditionally to treat oral infections. However, there is limited data validating its therapeutic properties and photochemistry. The aim of this study was to investigate the protective efficacy of the leaf and stem extracts of *C. bispinosa* against oral infections.

**Methods:**

The phenolic and tannin contents were measured using Folin-Ciocalteau method after extracting with different solvents. The minimum inhibitory concentrations (MIC) of the extracts were assessed using the microdilution method against fungal (*Candida albicans* and *Candida glabrata*) and bacterial (*Streptococcus pyogenes*, *Staphylococcus aureus* and *Enterococcus faecalis*) strains. The 2-diphenyl-1-picrylhydrazyl (DPPH) and ferric reducing power (FRP) models were utilised to assess the antioxidant potential of the extracts. Cytotoxicity of the leaf acetone extract was evaluated using the methylthiazol tetrazolium assay.

**Results:**

The methanol leaf extract had the highest phenolic content (113.20 mg TAE/g), whereas hexane extract displayed the highest tannin composition of 22.98 mg GAE/g. The acetone stem extract had the highest phenolic content (338 mg TAE/g) and the stem extract yielded the highest total tannin content (49.87 mg GAE/g). The methanol leaf extract demonstrated the lowest MIC value (0.31 mg/mL), whereas the stem ethanol extract had the least MIC value of 0.31 mg/mL. The stem methanol extract had the best DPPH free radical scavenging activity (IC_50,_ 72 µg/mL) whereas the stem ethanol extract displayed maximum FRP with absorbance of 1.916. The leaf acetone extract had minimum cytotoxicity with the lethal concentration (LC_50_) of 0.63 mg/mL.

**Conclusions:**

The results obtained in this study validated the protective effect of *C. bispinosa* against oral infections.

## Introduction

Oral infection is a global health burden and is prevalent mostly in low socioeconomic populations due to poor oral hygiene practises and lack of treatment facilities [1]. The oral cavity serves as the conducive habitat for a plethora of microorganisms comprising of archaea, protozoa, bacteria, fungi and viruses, with the most predominant group (96%) being bacteria belonging mainly to the phyla of Bacteroidetes, Fusobacteria, Proteobacteria, Actinobacteria, Firmicutes and Spirochaetes [2, 3]. A compendium of over 700 bacterial species have been reported to colonise the oral cavity [4]. Unfortunately, a large number of the oral microbiome are reported to be the causative agents of oral pathologies such as dental caries and periodontal [5]. Dental caries results to the irreversible damage to the enamel as the results of the metabolic activities of the oral pathogens, especially the cariogenic bacteria, which, through the acidic end-products of sucrose metabolism, decalcify minerals in the enamel [6]. Microbial dental plaque is not only the initiating factor for the occurrence of dental caries but also of periodontal disease. Periodontal disease is the severe inflammatory disease of the gums, which is characterised by the destruction of the attachment and supporting structures of the teeth. Periodontal infections can result in the teeth loss if left untreated [7].

Oxidative stress has been considered as one of the key etiological factors in etiopathogenesis of oral infections [8]. In response to oral infections caused by microorganisms and their toxins, the body consequentially produces excess free radicals, which are the metabolic by-products of oxygen: superoxide anion radical, hydrogen peroxide, perhydroxyl radical, hydroxyl radical, alkoxy radical, peroxyl radical etc., as an immune response [9]. The over-production of the free radicals often leads to the establishment of oxidative stress. The oxidative stress accelerates the progression and development of oral infections by triggering the oxidative damage on the structure functional biomolecules such as lipid and proteins [10]. Thus, oxidative stress has been strongly correlated with oral cancer and gangrenous stomatitis as well [8]. Antioxidantive compounds have abilities to suppress the activities of free radicals either by metal chelating, inhibition of enzymes activities or scavenging of free radicals, have therefore, become subjects of interest [11].

Several dentrifices and mouth rinses such as chlorhexidine, triclosan, fluorine, penicillin, erythromycin and amoxicillin have always been the choice for prevention and cure of oral infections [3]. This is due to their profound antimicrobial and antioxidant properties [12]. However, most of the oral microorganisms have acquired resistance towards most of the aliphatic antimicrobials [13]. Despite some degree of potency of the currently used medicine, their use has been a concern as most of them are costly and are strongly linked to be toxic and having other adverse effects such as hypersensitivity reaction, teeth staining, dorsum of the tongue, alteration in taste buds and the oral microbial flora [14–16]. These drawbacks have led to a continuous search for alternative remedy, especially of plant origin for development of antimicrobials that are effective and safe for use.

The plant-based phytochemicals such as phenols, alkaloids, tannins, and flavonoids to name but few, have been viewed as alternatives to complement the currently used dentrifices and antimicrobials in treating oral infections [17]. This is because they possess undeniable antimicrobial, anti-inflammatory and antioxidant properties [18]. Moreover, they have less, or no aftermath side effects, are inexpensive and easily available especially for people in the developing countries like South Africa.

*Carissa bispinosa* is one of the medicinal plants belonging to Apocynaceae [19]. The plant is widely distributed in South Africa, and it is valued because of its therapeutic potential and medicinal efficacies against diverse ailments including oral infections. Its stem and root are used for stimulation of male sex hormones in humans and for treatment of oral infections [20]. Kaunda and Zhang [21] have reported the presence of two terpenoids; ursolic acid and tritriacontane, which are well recognised for their antioxidant, anti-inflammatory and antimicrobial properties [22, 23]. Recently, the fruit extract of *C. bispinosa* has being reported to contain different types of flavonoids with antioxidant activity [24]. Nevertheless, there is still a lack limited scientific information on its phytoconstituents and their pharmacological activities.

The purpose of this study was to investigate the potential protective effect of phytoconstituents from the leaf and stem extracts of *C. bispinosa* by evaluating their antimicrobial activities against pathogens implicated in oral infection and their antioxidant and cytotoxic properties.

## Methodology

### Chemicals and media

The chemicals and culture media used were of analytic grade and were procured from Sigma-Aldrich, Whitehead scientific, Adcock-Ingram and Merck (Pty) Ltd. The water used in the experiments was distilled and autoclaved at 121 ^o^C for 15 min before use.

### Microbial strains

The fungal (*Candida albicans* and *Candida glabrata*) and bacterial (*Streptococcus pyogenes, Staphylococcus aureus* and *Enterococcus faecalis*) strains used in this study were the oral isolates obtained from Polokwane Hospital, National Health Laboratory Service (NHLS), South Africa.

### Plant collection

The stem and leaves of the plant were collected from the University of Limpopo Turfloop campus, in Limpopo province, South Africa (latitude − 23.885425 S, longitude E, altitude 1335 m) on July 8, 2019. The ethical clearance for the use of the plant was obtained from the research ethical committee at the University of Limpopo and the voucher specimens of the plant was deposited at the University of Limpopo Larry Leach herbarium (UNIN) for future reference (UNIN 1,220,078), the plant was identified by Dr Bronwyn Egan. The use of the plant extracts in this study were in alignment with the international guidelines [25]. The stem and leaves were washed with tap water to remove debris and soil particles and air-dried in the absence of light and heat to protect the structures of heat-sensitive compounds. The dry plant materials were milled to powder by an electric grinder (Sundy hamercrusher SDHC 150) and stored in a dark polyethylene plastic bag until further use.

### Extraction

The dried-milled powder (1 g) of each part (leaf and stem) was extracted with 10 mL of different solvents (from non-polar to polar) namely: hexane, chloroform, dichloromethane, ethyl acetate, acetone, ethanol, methanol, butanol, and water. The mixtures were shaken at a speed of 200 rpm for 20 min using a shaking incubator (New Brunswick Scientific Co., Inc). Afterwards, the extracts were filtered using Whatman No.1 filter papers. Subsequently, the solvents were evaporated using a fan. The water and butanol extracts were concentrated using a rotary evaporator (Buchi B-490) before drying them under a fan. Thereafter, the extracts were transferred into pre-weighed vials. The yield of each crude extracts was evaluated using the formula: yield = (A_o_ - A_1_), where A_o_ represents the final weight of the extract and the vial (mg) and A_1_ denotes the initial weight (mg) of the empty vial. The crude extracts were all reconstituted in acetone to the desired concentration (10 mg/mL).

## Quantitative analysis of classes of phytochemicals

### Total phenolic content

The total phenolic content of the plant extracts (in 70% aqueous acetone) was investigated using the method as described by Singleton et al. (1999). The total phenol composition of the extracts was expressed as mg of gallic acid equivalent per gram (mg GAE/g) of the extracts [26].

### Total tannin content

The total tannin content of the extracts was measured using the Folin-Ciocalteau method. The total tannin contents of the extracts were expressed in mg GAE/g of the extracts [26].

### Phytochemical analysis by chromatography

The phytocompounds from the leaf and stem extracts of *C. bispinosa* were analysed by Sciex Exion Liquid chromatography-mass spectrometry (LC-MS) equipment connected to Sciex X500R QTOF system and electron spray ionization detector according to Mpai et al. [27]. The separation was achieved using a Phenomenex Luna C18 column (2.5 μm, 100 × 2 mm particle size). The mobile phases (A and B) contained methanol and water supplemented with ammonium acetate (20 mM) respectively. The gradient elution procedure was done as follows: 0 min, 5% A and 95% B for 1 min, 5% A and 95% B for 22 min, 95% A and 5% B for 27 min, 95% A and 5% B for 27,10 min, 5% A and 95% B for 30 min, 5% A and 95% B. The flow rate (0.4 mL/min) was employed at 40 ◦C and the instrument was injected with 10 µL. The MS was run in a negative ion electrospray mode and nitrogen gas was utilised for desolvation. Ion source gas 1 and 2 were at 50 and 70 psi, respectively. The curtain gas was kept at 30 psi while CAD gas was at 7, ion source at 500 ◦C, spray voltage of used was 4500 and decluttering potential was − 80 V. Data acquisition and processing was performed using analyst software.

### Antimicrobial assay

#### Preparation of microorganisms

The stock cultures were prepared by inoculating the microbial isolates into 150 mL of Sabouraud dextrose broth for fungi and nutrient broth for bacteria and were incubated at 30 and 37 °C, respectively. The fungal and bacterial suspensions were adjusted to a density of 1 × 10^8^ CFU/mL using sterile distilled water.

### Bioautography assay

The antimicrobial activities of all extracts were investigated qualitatively against the fungal and bacterial isolates using a bioautography assay as outlined by Begue and Kline [28]. Twenty microliter of the extracts (10 mg/mL) was loaded on the thin layer chromatography (TLC) plates and the chromatograms were developed in 3 different mobile phases: benzene/ethanol/ammonium hydroxide [BEA] in a ratio of 9:1:0.1; chloroform/ethyl acetate/formic acid [CEF] (5:4:2) and ethyl acetate/methanol/water [EMW] (10:1.35:1). The bioautograms with clear zones indicated the growth inhibition by the extracts.

### Minimum inhibitory concentration (MIC) and total antimicrobial activity

The extracts were subjected to microdilution technique to determine their minimum inhibitory concentration (MIC) [29]. The MIC values of the extracts were regarded as the least concentration of the extracts to inhibit the fungal and bacterial growth. The total activities of all extracts were mathematically obtained by dividing the MICs by the mass extracted from 1 g of the *C. bispinosa* plant materials [30].

### Antioxidant activity

#### DPPH free radical scavenging assay

The DPPH radical scavenging activities of the obtained extracts were determined using the method described by Brand-Williams et al. [31], with some modifications. The percentage inhibition was measured using the formula: %Inhibition = (A_o_ – A_1_)/Ao) x 100, where A_o_ = absorbance of the control and A_1_ = absorbance of the sample.

#### Ferric reducing power (FRP) assay

The potential of the plant extracts to reduce potassium ferricyanide to potassium ferrocyanide was determined using the FRP as defined by Oyaizu [32], with some modifications. High absorbance of the extracts at 700 nm implies high ferric reductive potential. The blank was prepared in the same manner as the extracts using acetone.

### Cytotoxic activity

The cytotoxicity of the leaf acetone extract against human leukemia monocytic (THP-1) cell line was evaluated using MTT calorimetric assay as outlined by Mosmann [33], with some modifications. The THP-1 cell was maintained in a flask with Roswell Park Memorial Institute (RPMI 1640) medium supplemented with 10% foetal bovine serum (FBS). The percentage cell viability was measured by using the formula: %Cell viability = (A_o_ – A_1_) / A_o_) x 100, where A_o_ and A_1_ represent the absorbance readings of the untreated samples and treated samples, respectively.

### Preparation of ligands and receptor proteins

The three-dimensional (3D) structures of gentamicin and oleamide were downloaded from National Centre for Biotechnology Information (NCBI) PubChem compound database, and the proteins were retrieved from the Protein Data Bank (PDB) (www.rcsb.org). The target receptors used in this study are ATP-binding cassette (ABC) transporter (ID 6aal), and DNA gyrase A (ID 3g7b). The proteins were optimised to enhance effective docking by deleting water molecules, heteroatoms and other ligand groups prior to the addition of polar hydrogens using the Discovery Studio software version 4.1 [34].

### Molecular docking

Molecular docking was performed *in-silico* using AutoDock Vina. The binding sphere for 6aal (-0.302947, 21.052211 and − 23.891211 for x, y, and z centers) and 3g7b (-27.830552, -5.584207 and 8.598638 for x, y, and z centers) were identified from the active sites of the receptors using Discovery Studio 4.1. The binding affinities were estimated by assessing the binding scores of the ligand-receptor complexes using AutoDock Vina [35]. The lowest binding energy scores of the ligand-receptor complexes were selected as the best best-docked conformations. Thereafter, the docked complexes were imported to the Discovery Studio version 4.1 to visualise the generated 2 and 3 dimensional (2D and 3D) conformations [34].

### Data analysis

All the experimentations were triplicated, and the data obtained was expressed as mean ± standard-deviation. Data was analyzed using one way ANOVA and the values of *p* < 0.05 were considered as statistically significant.

## Results

### Extract yield

**Fig. 1:**
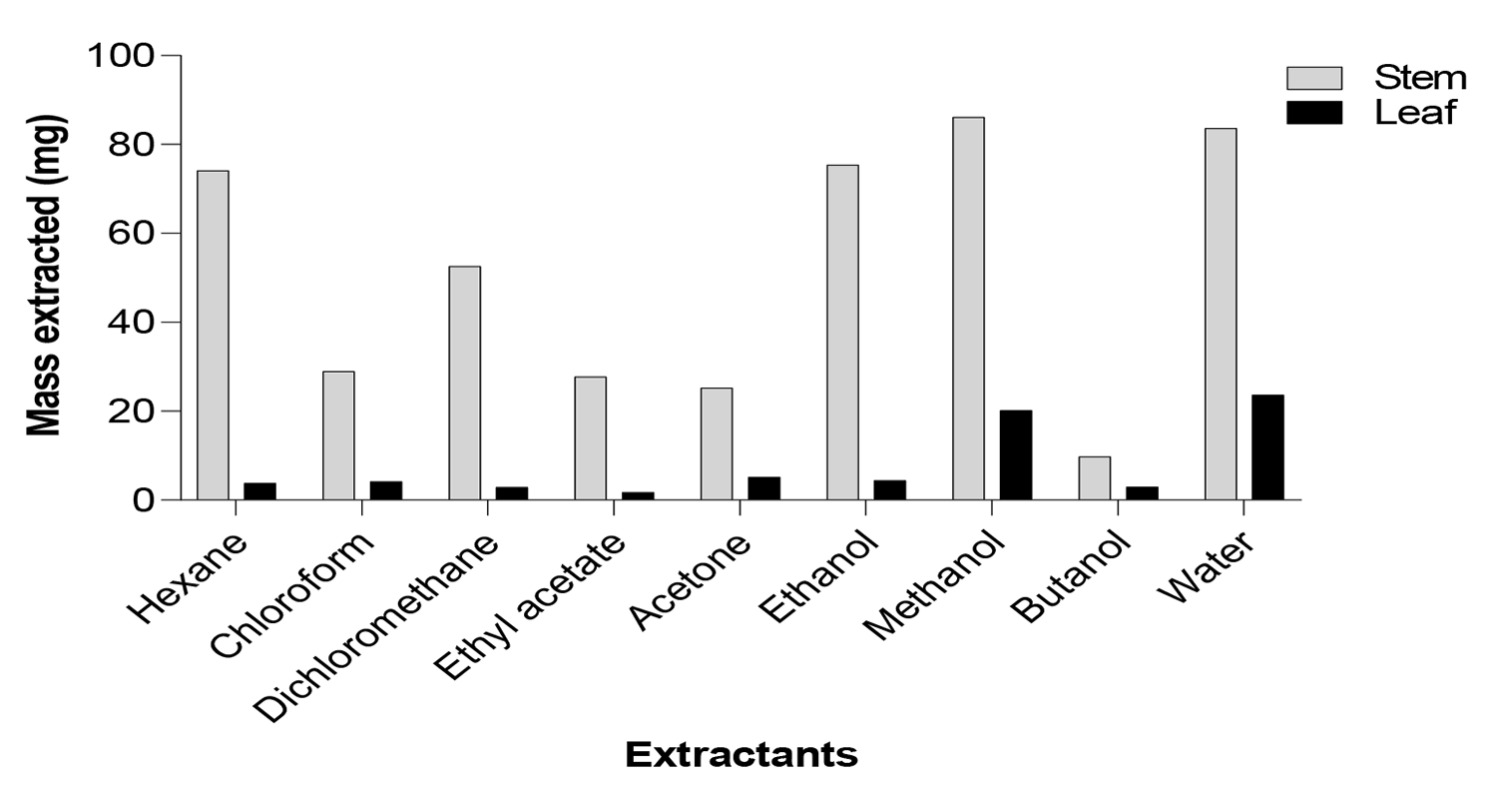
The yields of the leaf and stem extracts of *Carissa bispinosa* obtained using different solvents

Figure 1 illustrates the masses (mg) of the leaf and stem extracts obtained after extraction. The leaf extracts were relatively higher in comparison to the stem extracts. Furthermore, the polar solvents extracted the highest mass of phytochemicals.

### Analysis of phytocompounds of the extract by LC-MS

**Fig. 2:**
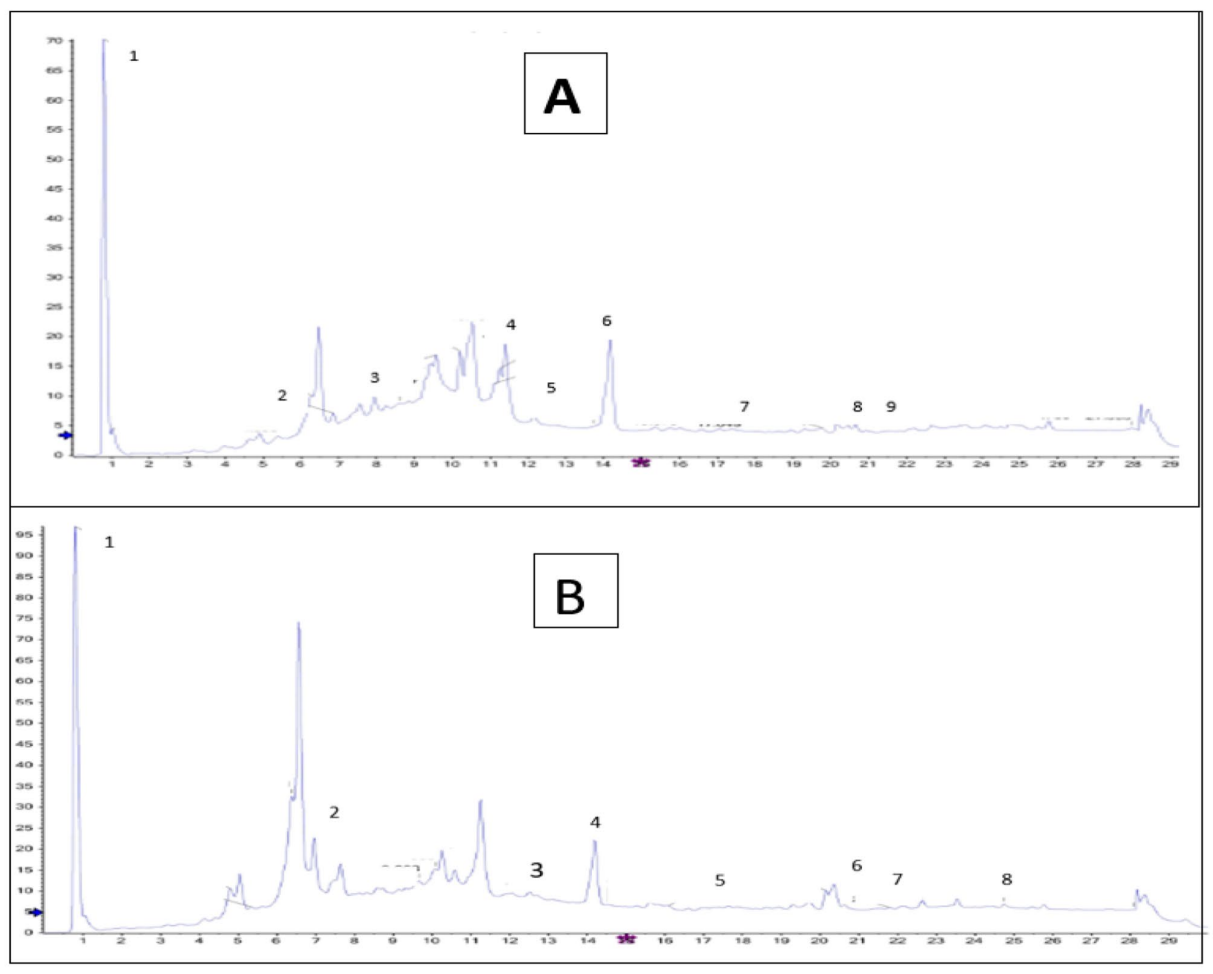
Chromatographic analysis of *C. bispinosa* methanol extracts. Total ion LC–MS/MS chromatograms of (**A**) the leaves and stem (**B**)

The phytocompounds and their retention time as revealed by the LC-MS analysis are shown in Fig. 2A (leaf extract) and 2B (steam extract) and Table 1 (leaf extract) and Table 2 (stem extract). The compounds that were detected in both leaf and stem extracts included among others oleamide, sorbitan monopalmitate and tributylamine.

**Table 1:** Compounds detected from the leaf methanol extract of *Carissa bispinosa* using Liquid chromatography-mass spectrometry (LC-MS)

Peak #	Rt (min)	Area %	MS (m/z)	MS-MS (m/z)	Molecular Formula	Tentative identification	Chemical class	Bioactivity
1	0.60	3.963e + 04	365.106 [M + H]+	185.1, 203.0,	C_19_H_24_OS_3_			
2	7.20	6.772e + 04	186.222 [M + H]+	74.1, 130.2	C_12_H_27_N	Tributylamine	Tertiary amines	
3	7.69	2.895e + 02	207.174 [M + H]+	67.1, 119.1, 189.0	C_14_H_22_O	2,4-Di-tert-butylphenol	Phenols	Antibacterial, antifungal [36]
4	11.24	9.879e + 03	527.246 [M + H]+	437.2, 527.1	C_23_H_38_N_6_O_4_S_2_			
5	12.77	7.628e + 03	434.289 [M + H]+	149.1, 236.9, 28.1	C_25_H_39_NO_5_	N-[1,1-Bis[(acetyloxy)methyl]-3-(4-octylphenyl)propyl]acetamide	Acetamide	
6	14.15	2.017e + 05	347.231 [M + H]+	121.0, 347.2	C_21_H_30_O_4_	Corticosterone	21-hydroxysteroids	
7	17.99	4.267e + 04	403.294 [M + H]+	403.3	C_22_H_42_O_6_	Sorbitan monopalmitate	Fatty acid esters	
8	21.10	2.236e + 04	194.115 [M + H]+	95.3	C_11_H_15_NO_2_	Isoprocarb	Carbamate ester	
9	22.37	5.995e + 06	282.279 [M + H]+	69.0, 83.1, 95.1, 107.1, 282.1	C1_8_H_35_NO	Oleamide	Fatty amide	Antibacterial [37, 38]

**Table 2:** Compounds detected from the stem methanol extract of *Carissa bispinosa* using Liquid chromatography-mass spectrometry

Peak #	Rt (min)	Area %	MS (m/z)	MS-MS (m/z)	Molecular Formula	Tentative identification	Chemical class	Bioactivity
1	0.60	3.963e + 04	365.106 [M + H]+	185.1, 203.0,	C_19_H2_4_OS_3_			
2	7.20	6.772e + 04	186.222 [M + H]+	74.1, 130.2	C_12_H_27_N	Tributylamine	Tertiary amines	
3	12.77	8.033e + 03	434.289 [M + H]+	81.1, 149.1, 258.1	C_25_H_39_NO_5_	N-[1,1-Bis [(acetyloxy) methyl]-3-(4-octylphenyl)propyl] acetamide	Acetamide	
4	14.15	2.017e + 05	347.231 [M + H]+	121.0, 347.2	C_21_H_30_O_4_	Corticosterone	21-hydroxysteroids	
5	17.99	4.267e + 04	403.294 [M + H]+	403.3	C_22_H_42_O_6_	Sorbitan monopalmitate	Fatty acid esters	
6	20.93	5.867e + 04	547.521 [M + H]+	67.1, 123.1, 408.4, 474.4	C_28_H_66_N_8_S			
7	22.37	5.995e + 06	282.279 [M + H]+	69.0, 83.1, 95.1, 107.1, 282.1	C_18_H_35_NO	Oleamide	Fatty amide	Antibacterial [37, 38]
8	25.54	1.933e + 06	537.395 [M + H]+	282.2, 299.3, 369.2, 537.5	C_40_H_56_	β-Carotene	Carotenoids	Antioxidant [39]

### Phenolic and tannin composition of the leaf and stem extracts

The phenolic and tannin constituents were quantified, and the findings are displayed in Table 3. The stem extracts exhibited higher total phenolic and tannin contents than the leaf extracts. The methanol leaf extract revealed the highest total phenolic content of 113.20 ± 10.4 mg GAE/g, whereas the acetone stem extract constituted the maximum phenolic composition (338 mg GAE/g). The least phenolic content of 29.21 ± 13.4 mg GAE/g was obtained with acetone leaf extract. Like the total phenolic content, the stem tannin content was higher than those from the leaf. The highest total tannin content was detected in hexane leaf extract (22.98 mg GAE/g); the ethanol stem extract yielded the highest total tannin content of 49.87 mg GAE/g.

**Table 3:** Total phenolic and tannin composition (mg of GAE/g) of the leaf and stem extracts of *Carissa bispinosa*

Solvent	Phenolic content			Tannin content	
	Leaf extract	Stem extract	Leaf extract		Stem extract
Hexane	29.98 ± 18.0 ^a^	216 ± 30.6 ^b^	22.98 ± 2.2 ^a,c^		47.29 ± 2.8 ^e^
Dichloromethane	70.41 ± 34.8 ^a,b^	50 ± 18.2 ^a^	8.36 ± 1.0 ^b^		8.89 ± 0.7 ^a, b^
Ethyl acetate	69.64 ± 25.5 ^a,b^	36 ± 7.8 ^a^	16.64 ± 2.6 ^c,d,e^		6.79 ± 1.7 ^a^
Acetone	29.21 ± 13.4 ^a^	338 ± 14.9 ^c^	15.04 ± 2.3 ^b,d,e^		47.65 ± 2.5 ^e^
Ethanol	33.79 ± 11.3 ^a^	216 ± 59.4 ^b^	13.46 ± 0.7 ^b,d^		49.87 ± 5.0 ^e^
Methanol	113.20 ± 10.4 ^a^	163 ± 26.8 ^b^	9.35 ± 5.0 ^b,f^		28.07 ± 7.5 ^c,d^
Butanol	40.01 ± 7.6 ^b^	31 ± 10.7 ^a^	10.65 ± 1.9 ^b,d^		12.23 ± 3.3 ^a,b^
Chloroform	30.23 ± 19.3 ^a^	46 ± 17.6 ^a^	15.37 ± 1.2 ^d,e,f^		18.21 ± 4.4 ^b,c^
Water	94.74 ± 12.6 ^b^	244 ± 17.6 ^b^	20.01 ± 1.4 ^a,e^		38.68 ± 2.0 ^d,e^

In each column, average values with the same letters have a statistical insignificant difference (Tukey’s multiple comparison test)

### Antimicrobial activity of the leaf and stem extracts

#### Bioautography assay of the leaf and stem extracts

**Fig. 3:**
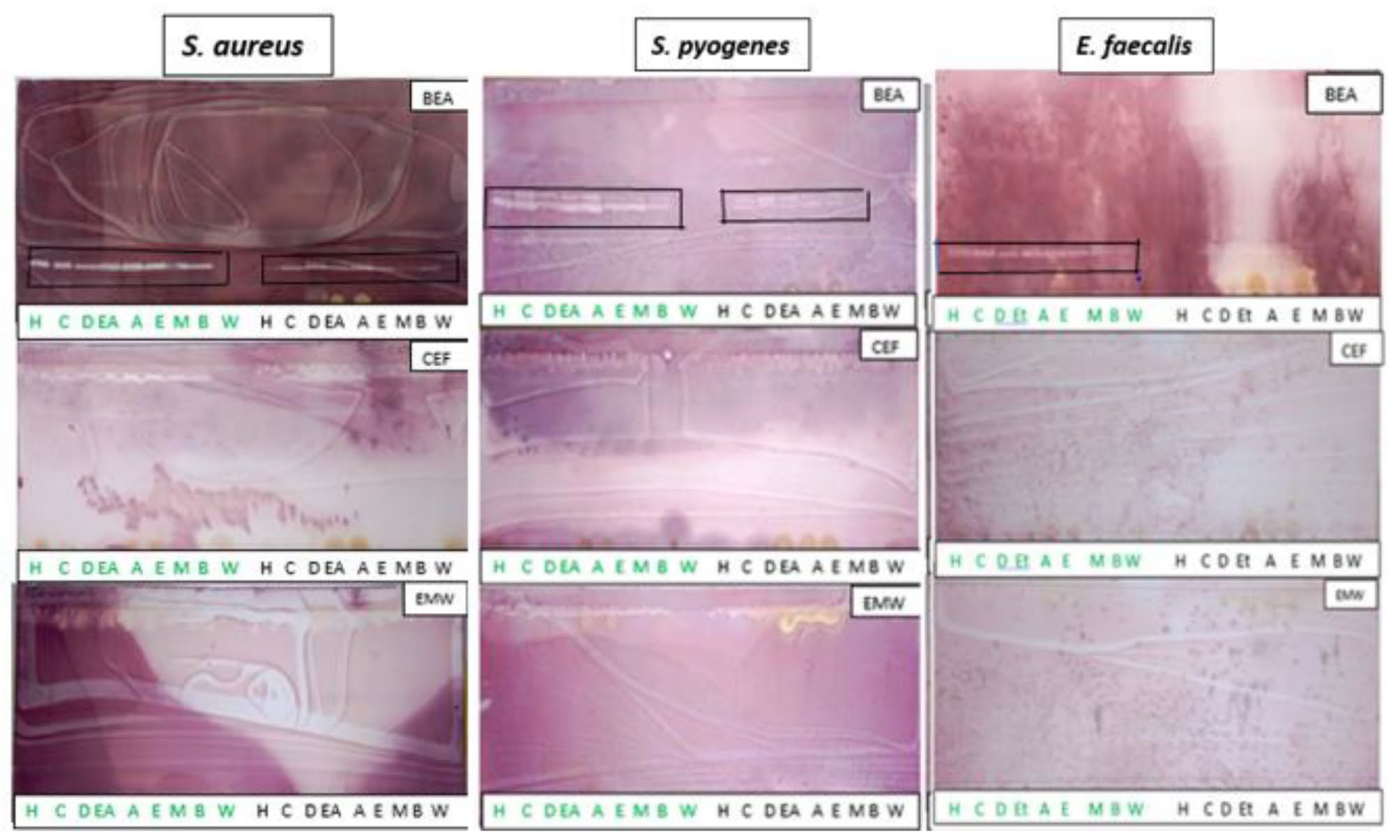
Bioautograms of different *Carissa bispinosa* leaf and stem extracts against *Staphylococcus aureus, Streptococcus pyogenes and Enterococcus faecalis* developed in (benzene/ethanol/ammonium hydroxide 9:1:0.1), (chloroform/ethyl acetate/formic acid 5:4:2) and (ethyl acetate/methanol/water 10:1.35:1) mobile phases. Key: (H) hexane, (C) chloroform, (D) dichloromethane, (EA) ethyl acetate, (A) acetone, (E) ethanol, (M) methanol, (B) butanol and (W) water. The green markings represent the leaf extracts while the black represent those of the stem

Bioautography assay was performed to determine the antimicrobial efficacies of the leaf and stem extracts against microorganisms implicated in oral infections. The antibacterial activity of the leaf and stem extracts were the marked areas, representing the zones of bacterial inhibition on the chromatograms (Fig. 3). Antibacterial activity was observed on all bacterial strains with BEA chromatograms being the only one that demonstrated the activity. Generally, the leaf extracts showed better inhibitory activities than the stem extracts. All the leaf extracts, except for the water extract, displayed antibacterial activity against the tested pathogens (*S. aureus, E. faecalis*, and *S. pyogenes*).

**Fig. 4:**
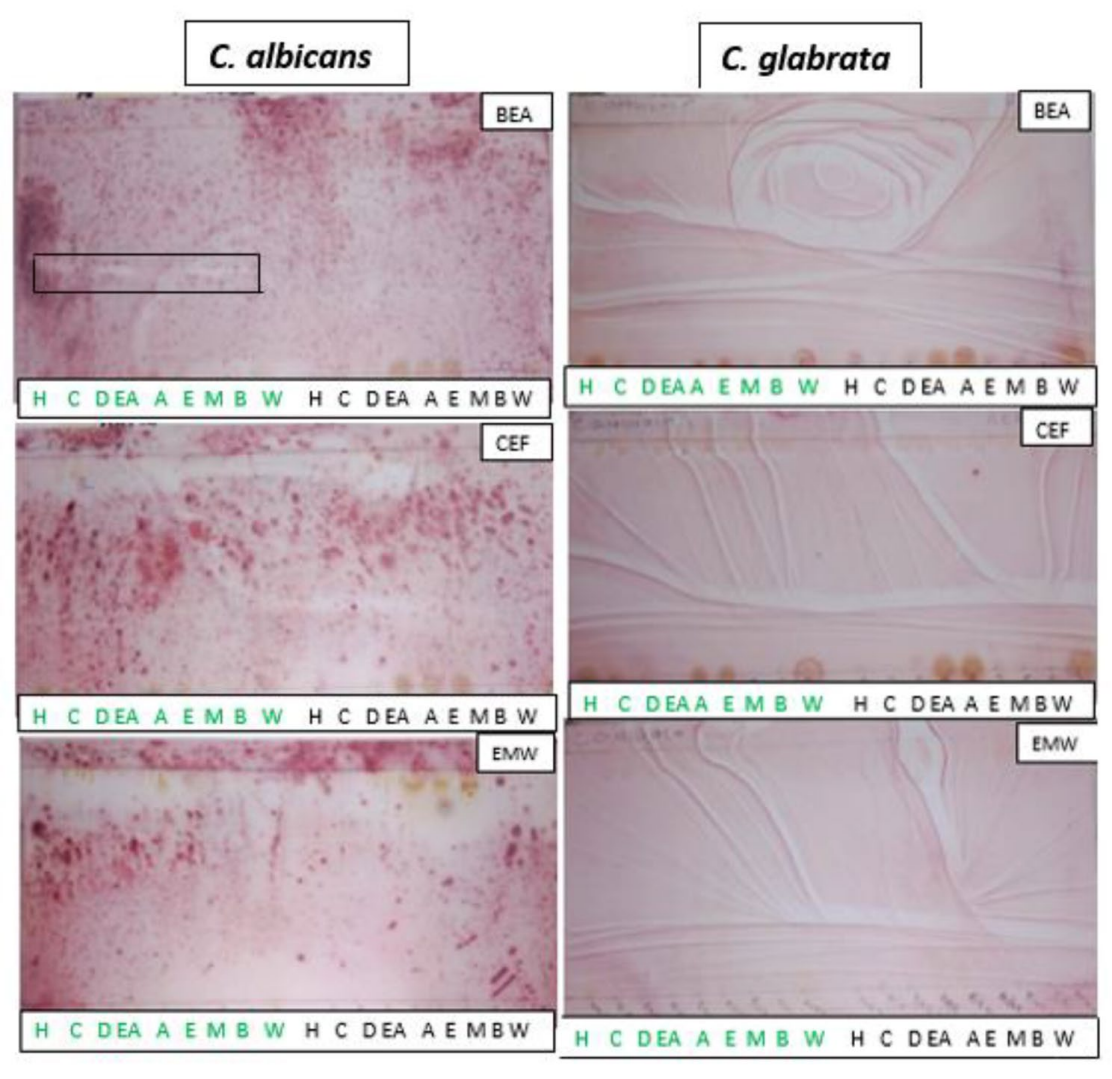
Bioautograms of different *Carissa bispinosa* leaf and stem extracts against *Candida albicans* and *Candida glabrata* developed in (benzene/ethanol/ammonium hydroxide 9:1:0.1), (chloroform/ethyl acetate/formic acid 5:4:2) and (ethyl acetate/methanol/water 10:1.35:1) mobile phases. Key: (H) hexane, (C) chloroform, (D) dichloromethane, (EA) ethyl acetate, (A) acetone, (E) ethanol, (M) methanol, (B) butanol and (W) water. The green markings represent the leaf extracts while the black represent those of the stem

The antifungal activity of the different *C. bispinosa’s* leaf and stem extracts on *C. albicans* and *C. glabrata* are illustrated in Fig. 4. Faint inhibition zones were only observed on the BEA chromatogram sprayed with *C. albicans* culture, indicative of antifungal activity of the leaf extract against this strain. There was no antifungal activity observed on *C. glabrata.*

### MICs of the leaf and stem extracts

#### MICs of the leaf extracts

The broth microdilution method was implemented to quantitatively determine the antibacterial and antifungal activities of the leaf extracts. Methanol leaf extract displayed the lowest MIC value (0.31 mg/mL), making it the most active across the microorganisms. Ethyl acetate and butanol leaf extracts illustrated the highest MIC value (≥ 1.25 mg/mL). *C. albicans* was the most susceptible pathogen and was inhibited by an MIC of 0.31 mg/mL while *C. glabrata* was the most resistant (≥ 1.25 mg/mL) (Table 4).

**Table 4:** MIC values (mg/mL) of the *Carissa bispinosa* leaf extracts, gentamicin and amphotericin-B against bacterial and fungal pathogens

Microorganism	H	C	D	EA	A	E	M	B	W	Gen/Amp
*S. aureus*	1.25	0.63	0.63	2.5	2.5	0.63	0.31	2.5	0.31	0.15
*S. pyogenes*	1.25	1.25	0.63	1.25	1.25	0.63	0.31	1.25	1.25	0.08
*E. faecalis*	2.5	2.5	2.5	2.5	0.63	1.25	0.63	1.25	1.25	0.15
*C. albicans*	0.63	0.63	1.25	1.25	0.63	0.31	0.31	1.25	0.31	0.08
*C. glabrata*	2.5	2.5	2.5	1.25	1.25	2.5	2.5	2.5	1.25	0.08

Key: (H) hexane, (C) chloroform, (D) dichloromethane, (EA) ethyl acetate, (A) acetone, (E) ethanol, (M) methanol, (B) butanol, (W) water, (Gen/Amp) gentamicin/ amphotericin-B

Fungal (*Candida albicans* and *Candida glabrata*) and bacterial (*Streptococcus pyogenes, Staphylococcus aureus* and *Enterococcus faecalis*) strains

## Minimum inhibitory concentration of the stem extracts

The stem extracts were analysed to ascertain their MICs (Table 5). The ethanol extract had the lowest MIC of 0.31 mg/mL, while chloroform displayed the highest MIC (≥ 1.25 mg/mL). The microorganism that was mostly susceptible to the least concentration of the extract was *S. pyogenes, C. glabrata* was the most resistant strain and was inhibited by MIC (≥ 1.25 mg/mL).

**Table 5:** MIC values (mg/mL) of the *Carissa bispinosa* stem extracts, gentamicin and amphotericin-B against bacterial and fungal pathogens

Microorganism	H	C	D	EA	A	E	M	B	W	Gen/Amp
*S. aureus*	1.25	5	1.25	2.5	1.25	0.31	2.5	2.5	2.5	0.31
*S. pyogenes*	5	1.25	0.63	0.63	0.31	0.63	1.25	0.63	1.25	0.15
*E. faecalis*	1.25	2.5	2.5	1.25	1.25	0.63	1.25	2.5	1.25	0.15
*C. albicans*	2.5	2.5	2.5	5	0.63	0.31	0.63	2.5	2.5	0.31
*C. glabrata*	5	5	2.5	2.5	1.25	1.25	2.5	2.5	2.5	0.15

Key: (H) hexane, (C) chloroform, (D) dichloromethane, (EA) ethyl acetate, (A) acetone, (E) ethanol, (M) methanol, (B) butanol and (W) water. Fungal (*Candida albicans* and *Candida glabrata*) and bacterial (*Streptococcus pyogenes, Staphylococcus aureus* and *Enterococcus faecalis*) strains

### Total antimicrobial activities of the leaf and stem extracts

#### Total antimicrobial activities of the leaf extracts

The total antimicrobial activities of the leaf extracts were also determined; and the results are demonstrated in Table 6. The peak overall antimicrobial activity of 278 mL/g was observed when the methanol extract was utilised, whereas butanol extract revealed the lowest antimicrobial action (3.9 mL/g).

**Table 6:** Total antimicrobial activities (mL/g) of the *Carissa bispinosa* leaf extracts against bacterial and fungal pathogens

Microorganism	H	C	D	EA	A	E	M	B	W
*S. aureus*	59.3	46.0	83.5	11.1	10.1	119.7	278.1	3.9	270
*S. pyogenes*	59.3	23.2	83.5	22.2	20.2	119.7	278.1	7.8	67.0
*E. faecalis*	29.6	11.6	21.0	11.1	26.4	60.3	136.8	7.8	67.0
*C. albicans*	117.6	46.0	42.1	22.2	40.2	243.2	278.1	7.8	270
*C. glabrata*	29.6	11.6	21.0	22.2	20.2	30.2	34.5	3.9	67.0

Key: (H) hexane, (C) chloroform, (D) dichloromethane, (EA) ethyl acetate, (A) acetone, (E) ethanol, (M) methanol, (B) butanol and (W) water

Fungal (*Candida albicans* and *Candida glabrata*) and bacterial (*Streptococcus pyogenes, Staphylococcus aureus* and *Enterococcus faecalis*) strains

#### Total antimicrobial activities of the stem extracts

Table 7 displays the total antimicrobial activities of stem extracts. Methanol stem extract revealed the maximum total antimicrobial activity (32.06 mL/g) while ethyl acetate demonstrated the least total antimicrobial activity of 0.36 mL/g.

**Table 7:** Total antimicrobial activities (mL/g) of the *Carissa bispinosa* stem extracts against bacterial and fungal pathogens

Microorganism	H	C	D	EA	A	E	M	B	W
*S. aureus*	3.04	0.84	2.32	0.72	4.16	14.19	0.08	1.2	9.48
*S. pyogenes*	0.76	3.36	4.60	2.86	16.77	6.98	16.16	4.76	18.96
*E. faecalis*	3.04	1.68	1.16	1.44	4.16	6.98	16.16	1.2	18.96
*C. albicans*	1.52	1.68	1.16	0.36	8.25	14.19	32.06	1.2	9.48
*C. glabrata*	0.76	0.84	1.16	0.72	4.16	3.52	8.08	1.2	9.48

Key: (H) hexane, (C) chloroform, (D) dichloromethane, (EA) ethyl acetate, (A) acetone, (E) ethanol, (M) methanol, (B) butanol and (W) water

Fungal (*Candida albicans* and *Candida glabrata*) and bacterial (*Streptococcus pyogenes, Staphylococcus aureus* and *Enterococcus faecalis*) strains

### Antioxidant activities of the leaf and stem extracts

#### DPPH scavenging activities of the leaf and stem extracts

The leaf and stem extracts were analysed for their antioxidant potency using DPPH free radical scavenging assay and the results are demonstrated in Table 8. Methanol and ethanol leaf extracts had a highest scavenging activity with an IC_50_ value of 95 µg/mL).

**Table 8:** IC_50_ (µg/mL) of the *Carissa bispinosa* leaf and stem extracts against DPPH.

Extractant	Leaf	Stem
Hexane	–	198
Dichloromethane	–	–
Ethyl acetate	–	–
Acetone	206	–
Ethanol	95	105
Methanol	95	72
Butanol	–	221
Water	75	172
Ascorbic acid	11.6	11.6

Key: (–) Not determined

### Ferric reducing power of the leaf and stem extracts

**Fig. 5:**
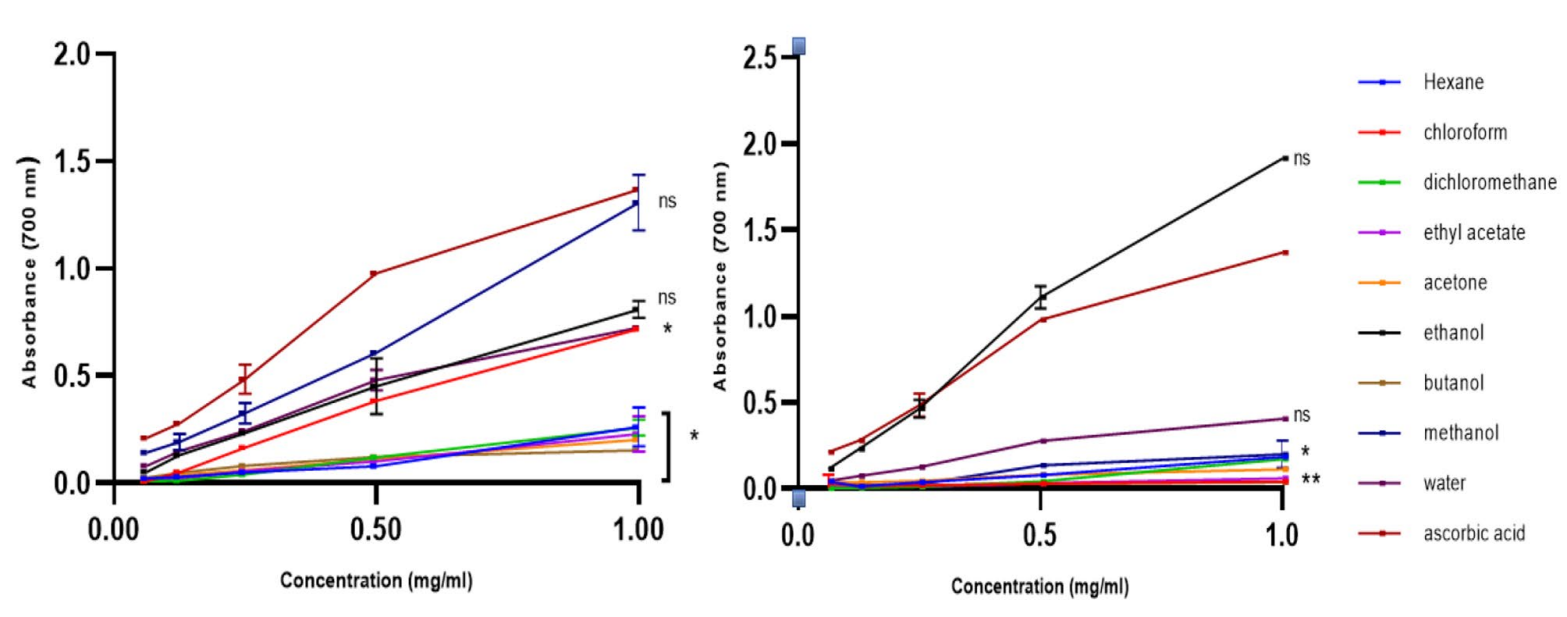
Ferric reducing power of the *Carissa bispinosa* leaf (left) and stem (right) extracts of *Carissa bispinosa*. The results are expressed as mean ± SD of three replicates. One-way ANOVA was used for statistical analysis (*p* < 0.05). Key: (*) significant difference, (ns) non-significant difference

Figure 5 illustrates the reducing power of the leaf and stem extracts. The leaf extracts demonstrated ferric reducing ability in a concentration dependant manner; the reducing power of the leaf extracts improved with the increase in concentration. Polar extracts (methanol leaf extract and ethanol stem extract) exhibited the highest reducing power with the maximum absorption of 1.308 and 1.916 respectively. However, L-ascorbic acid (control) revealed better activity than all leaf extracts and slightly lesser activity than the ethanol stem extract, which demonstrated the highest FRP with the absorption of 1.916.

### Cytotoxic effects the leaf acetone extract

The cytotoxic effect of the leaf acetone extract was evaluated using MTT assay. The extract exhibited a dose dependant cytotoxic effect with the LC_50_ of 0.63 mg/mL.

### Binding scores of docked complexes

The ligands (oleamide and gentimicin) were docked with 6aal and 3g7b proteins to estimate their binding affinities. Oleamide exhibited the lowest binding score of -5.6 kcal/mol against 6aal and − 4.2 kcal/mol against 3g7b. Gentimicin had lower binding scores (-7.7 kcal/mol against 6aal and − 5.8 kcal/mol on 3g7b) than oleamide.

### Interactions of the docked complexes

**Fig. 6:**
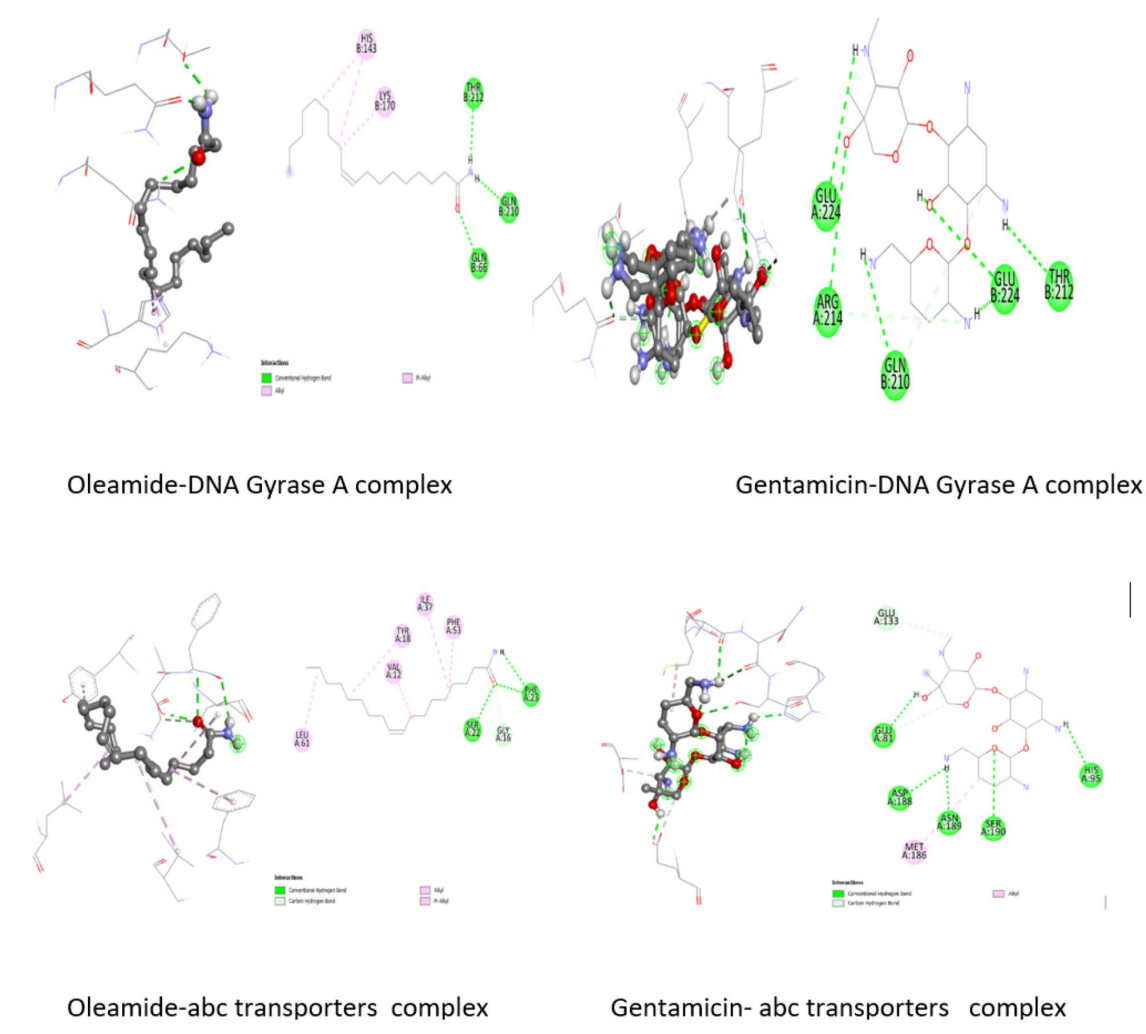
The 2D and 3D structures of the docked complexes

The interactions of the identified oleamide and receptors (6aal and 3g7b) were also investigated and the results are shown in Fig. 6. Oleamide formed two hydrogen bonds with SER22 and PHE23 of 6aal and the interactions were further strengthened by alkyl (VAL12, TYR18, ILE37, PHE53 and LEU61). It also revealed 3 hydrogen interactions (GLN66, GLN210 and THR212) and 2 alky bonds (HIS143 and LYS170) with 3g7b. The gentimicin-6aal complex exhibited 5 hydrogen bonds (GLU81, HIS95, ASP188, ASN189 and SER190), covalent bond (GLU133) and alkyl bond (MET186). Moreover, gentamicin-3g7b complex produced 5 hydrogen interactions (GLN210, THR212, ARG214, GLUA224 and GLUB224) and van der Waals forces.

## Discussion

Plants are recognised to be prolific producers of various phytochemicals of pharmacological importance. To obtain better quantity and bioactivity of plant-based extracts, selection of proper solvents is crucial. Therefore, in this study different solvents were used to extract the phytochemicals. Methanol and water illustrated the highest abilities to extract large number of phytochemicals in both leaf (methanol) and stem (water) parts of *C. bispinosa*, implying that these polar solvents may be effectively used for extracting phytochemicals in this plant. The ability of methanol to extract the highest mass of metabolites correlates with the results observed by Masoko and Eloff [40] and that of water, which was also shown by Bouhafsoun et al. [41].

The pharmacological properties of plant materials rely on the presences and quantity of its phytochemicals. In this study, the two classes of phytochemicals namely phenolic and tannin were quantitatively analysed, and the stem extracts revealed higher total phenolic and tannin compositions than the leaf extracts. Literature has reported that a high phenolic content often correlates with a high antioxidant action [42–46]. Therefore, *C. bispinosa* has potential applicability as antioxidant sources. Furthermore, The LC-MS identified compounds such as oleamide, which is recognised for its antimicrobial activity and antioxidant compound (β-carotene) are perceived to contribute to the bioactivity of this plant [37, 38, 47].

In oral diagnosis, the use of effective antimicrobial agents is paramount to reduce the burden caused by oral pathogens. Plants have been recognised as having profound efficacies against oral pathogens [48]. Thus, in this study, the qualitative antimicrobial activity illustrated that all leaf extracts, except for the water extract, demonstrated antimicrobial activities against all bacterial pathogens and the fungal strain *C. albicans*. However, *C. glabrata* was resistant to all extracts. Most of the inhibition zones were observed on the BEA chromatograms, suggesting that the antimicrobial compound(s) are probably non-polar.

The antimicrobial activity was also quantitatively assessed to ascertain the MICs of the plant extracts. For the leaf extracts, methanol extract presented the least average MIC, making it the most activity across the microorganisms. Moreover, the ethanol stem extract displayed the lowest average MIC. Plant extracts are regarded to possess noteworthy antimicrobial activities when the MIC value is less than 1 mg/mL [49]. Therefore, the methanol leaf and ethanol stem extracts can be generally regarded as noteworthy and can serve as good sources of antimicrobial compounds for treatment of oral pathogens. Moreover, the methanol leaf and stem extracts exhibited the highest total antimicrobial activities, suggesting that one gram of the leaf and stem materials can be diluted to 201.10 and 16.11 mL/g, respectively and still demonstrate inhibitory effects against the susceptible oral microbial strains [28].

Free radicals formed in the body as the result of environmental and biological factors cause oxidative stress, which often disturbs the normal redox state, consequently triggering oral infections. Thus, the search for antioxidants which can effectively nullify the progression of oral infections caused by oxidative stress have become an important aspect. In this study, methanol and ethanol extracts displayed high antioxidant potential by scavenging DPPH radicals and reducing ferricyanide, supporting the ability of methanol to extract antioxidants as observed by Ebrahimzadeh et al. [50]. The IC_50_ values of the both the ethanol and methanol leaf extracts was 95 µg/mL which is considered strong according to this range: very strong (IC_50_ < 50 µg/mL), strong (50 ≤ IC_50_ < 100 µg/mL), moderate (100 ≤ IC_50_ < 150 µg/mL), and low (IC_50_ > 150 µg/mL) [51]. The antioxidant activities observed were attributed to the synergistic effect of the identified phytochemicals. Moreover, there was a positive correlation between antioxidant activity and the total phenolic content, implying that the phenolic compounds could have played a major role to the observed antioxidant activity (unpublished).

High toxicity levels of plant extracts and compounds accounted for over 54% of failures in the preclinical phases in drug discovery [52]. The International Organization for Standardization consider cell viability above 80% as non-cytotoxic; 60–80% as weak; whereas 40–60% moderate and below 40% as strong cytotoxicity [53]. The percentage cell viability of the leaf acetone ranged of between 51 and 93%. Therefore, the extract has a high level of biosafety as it exhibited moderate to nontoxic effects. However, it is advisable to use low concentrations to avoid the occurrence of devastating effects that the extract might cause at high concentrations. Muleya et al. [20] have reported the traditionally used and effectiveness of *C. bispinosa`s* roots in to treating toothache. Therefore, the observed efficacies of the plant extracts against the oral pathogens and free radicals and the high biosafety level, suggests that these plant parts can serve as alternatives to the use of the roots for conservation purposes of this plant [54].

The molecular docking approaches was used to predict and elucidate the mode of inhibition observed the *in-vitro* antimicrobial activity in this study. The target receptors used in this study are ABC transporter (ID 6aal), and DNA gyrase A (ID 3g7b). DNA gyrase A plays a pivotal role in replication and super-coiling of microbial DNA molecules [55] whereas ABC transporters are membrane proteins that facilitate import and exports of molecules such as nutrients across the microbial membranes and are responsible for adenosine triphosphate (ATP) hydrolysis [56]. Thus, the two proteins are the main targets during antimicrobial drug design and development.

The lower binding energy score values observed in this study indicated the decent fitness of the oleamide in the binding pocket of both receptors, implying that the ligand has binding affinities with the receptors and has established good interactions with them. Moreover, this confirmed oleamide to have contributed to the antimicrobial activity observed in this study [57]. However, gentamicin illustrated better binding affinities against both receptors in comparison to oleamide, implying better inhibitory action. Nevertheless, it should be noted that the profound antimicrobial activity of the extracts might have been owed to the synergistic effect of the compounds within the extracts.

The antimicrobial activities of the ligands are not only governed by the binding energy but more precisely, by type of interactions formed between the ligand-receptor complexes [58]. Hydrogen, alkyl, covalent and van der Waals bonds were formed between the ligands and the target proteins, suggesting their involvement and effects during the observed antimicrobial activity in this study. H-bonds are relevant in ligand-target receptor interactions as they stabilise the ligand-receptor complexes, consequently leading to inhibition of microbial growth [59]. Alkyl bonds are covalent bonds which mostly strengthen the formed complexes. Covalent bonds are strong, hence the ligand-receptor complexes they form tend to be permanent [60]. Therefore, based on the results from the molecular docking study, the extracts from *C. albicans* have potential to treat oral infections by inhibiting and/or killing microorganisms implicated in the oral infections.

## Conclusion

The study evaluated the phytochemistry, antioxidant, antimicrobial and cytotoxic properties of the leaf and stem of *C. bispinosa*. The leaf extracts exhibited low MIC values against the tested oral pathogens. The same extracts revealed promising antioxidant activities, indicating their potential protective effects against oral infections caused by oxidative stresses. Moreover, the leaf extract revealed minimal cytotoxicity, especially at low concentration (93% cell viability at a concentration of 0.25 mg/mL), suggesting its possession of high level of biosafety. Furthermore, the molecular docking study predicted oleamide to exert antimicrobial activity by interacting with the targeted proteins through diverse bonds. The observed bioactivities were attributed to the synergistic effect of the identified phytochemicals within the extracts. Further research may focus on isolation and characterization of the antimicrobial compound(s) and in vivo studies.

## Data Availability

The datasets used and analysed during the current study is available from the corresponding author on reasonable request. The authors declare no conflict of interest.

## Abbreviations

*C. bispinosa*: *Carissa bispinosa*
*S. aureus*: *Staphylococcus aureus*
*S. pyogenes*: *Streptococcus pyogenes*
*E. faecalis*: *Enterococcus faecalis*
*C. albicans*: *Candida albicans*
*C. glabrata*: *Candida glabrata*
UNIN: University of Limpopo Larry Leach herbarium
TLC: Thin layer chromatography
RPMI 1640: Roswell Park Memorial Institute
THP-1: leukemia monocytic
DPPH: 2-diphenyl-1-picrylhydrazyl
FRP: Ferric reducing power
CFU: Colony forming units
MIC: Minimum inhibitory concentration
DMSO: Dimethyl sulfoxide
INT: Piodonitrotetrazodium violet
BEA: Benzene/ethanol/ammonium hydroxide
CEF: Chloroform/ethyl acetate/formic acid
EMW: Ethyl acetate/methanol/water
H: Hexane
C: Chloroform
D: Dichloromethane
EA: Ethyl acetate
A: Acetone
E: Ethanol
E: Methanol
M: Butanol
W: Water
AVG: Average
AA: Ascorbic acid
NHLS: National Health Laboratory Service
LC-MS: Liquid chromatography-mass spectroscopy
ATP: Adenosine triphosphate
ABC: ATP-binding cassette
6aal: ATP transporter
3g7b: DNA gyrase A
